# Shoulder pain prevalence by age and within occupational groups: a systematic review

**DOI:** 10.1186/s40945-021-00119-w

**Published:** 2021-11-04

**Authors:** Christopher J. Hodgetts, Charlotte Leboeuf-Yde, Amber Beynon, Bruce F. Walker

**Affiliations:** 1 Discipline of Chiropractic, College of Science, Health, Engineering and Education, Murdoch University, Perth, Western Australia Australia; 2 Centre for Molecular Medicine and Innovative Therapeutics, Health Futures Institute, Murdoch University, Perth, Western Australia Australia; 3 Institute of Regional Health Research, University of Southern Denmark, Odense C, Denmark

**Keywords:** (MeSH): shoulder pain, Prevalence, Occupational injuries, Age groups, Rotator cuff, Shoulder impingement syndrome

## Abstract

**Background:**

Shoulder pain was previously shown to diminish in older populations and it was suggested that this could be explained by reduced usage with age. Our objectives were to investigate if estimates of shoulder pain continue to increase after the age of 50 in working populations and to compare these estimates in physically demanding occupations with sedentary occupations.

**Methods:**

A systematic review of retrospective, cross-sectional, prospective, or longitudinal.

studies reporting prevalence or incidence of non-specific shoulder pain in occupational groups stratified by age. Searches were conducted in PubMed, Scopus, and CINAHL from inception until January 2020. Study characteristics and prevalence estimates stratified by age were extracted. Two reviewers independently performed a critical analysis of the included studies to determine their validity and risk of bias.

**Results:**

Twenty studies with a total of 40,487 participants and one study of a clinical data base were included and assigned a direction of the estimates for shoulder pain as either ‘increasing’, ‘remaining stable’ or ‘decreasing’ past the age of 50. Shoulder pain generally increased past 50, with 16 of the 21 included studies reporting higher estimates/odds ratios in older participants. In the more physically active occupations over 50, the estimates increased in 14 of the 18 samples compared to only two of the four involving sedentary occupations.

**Conclusions:**

Shoulder pain prevalence remains common in workers beyond the age of 50. Prevalence continues to increase in physically demanding occupations. Clinicians should consider factors of occupation when managing shoulder pain.

**Trial registration:**

**PROSPERO** (CRD42019137831).

**Supplementary Information:**

The online version contains supplementary material available at 10.1186/s40945-021-00119-w.

## Introduction

Shoulder pain is a common complaint, with point prevalence and lifetime estimates as high as 26 and 67%, respectively [1]. It is often associated with rotator cuff lesions [2, 3], but the causal relationship remains a point of debate [3–6]. Vincent et al. [7] reported in a systematic review of the general population that the prevalence of rotator cuff degeneration increases linearly with age. However, the same review found that shoulder pain does not continue in a similar linear fashion after 60–65 years of age but that it rather diminishes. Thus, the authors speculated that this could be linked to diminished work demands in later life. In support of this theory, a large study of several occupational groups reporting estimates for the age groups 40–49, 50–59, 60–69 and 70–74 showed that the 1-yr prevalence estimates continued to increase significantly but that this might be particularly noticeable in the ‘blue-collar’ rather than in the ‘white-collar’ occupations [8]. This research, therefore, suggests that continued activity as well as type of occupation are important contributors to the continued experience of shoulder pain, rather than a general reduction of pain in the older age groups.

In other words, the disparity between the prevalence of rotator cuff degeneration and shoulder pain in these older age groups could have multiple explanations. Firstly, it is possible that rotator cuff degeneration itself is not responsible for the shoulder pain. Secondly, it is possible that degeneration plays a role in the production of musculoskeletal pain, which is exacerbated in combination with certain levels of activity.

In studies of the general population that include elderly people, a large proportion would be retired or have reduced their physical activities at work and at home, and the physically active would likely be a smaller group making a link between work and pain in the total sample difficult to detect. Therefore, it is necessary to estimate the prevalence of shoulder pain in specific age groups whilst taking into account their work status. For this reason, we chose to undertake a systematic review investigating whether occupational activities, generally, influence shoulder pain prevalence in older adults. Accordingly, the purpose of this systematic review was to explore the following two questions:

- Does the prevalence of shoulder pain continue to increase after the age of 50 in those who are still work active?
- If so, is there a difference between those whose occupations are physically demanding from those who have sedentary occupations?

## Methods

### Design

This systematic review is registered with PROSPERO (CRD42019137831) and was completed in accordance with the Preferred Reporting Items for Systematic Reviews and Meta-analyses (PRISMA) guidelines (see Additional file 1) [9].

### Search strategy

Searches were performed using PubMed, Scopus, and CINAHL from inception to January of 2020 and the search strategy is found in Additional file 2. The titles and abstracts were screened independently by two reviewers (CH, AB) to identify potentially relevant articles. The reviewers then independently screened these full-text articles for inclusion. A final screening procedure on full-text articles was performed with the help of another reviewer (CLY) until consensus was reached. Reference lists were also searched to identify any additional studies.

### Eligibility criteria

We were interested in studies that reported data specifically for different occupational groups and provided prevalence estimates or odds ratios for increasing age brackets, to make it possible to compare age stratified shoulder pain prevalence estimates in white and blue collar workers.

#### Inclusion criteria

- Studies on the prevalence or incidence of non-specific shoulder pain, including rotator cuff disease or subacromial bursitis
- Study populations including occupational groups, or the general population reported by occupational groups, workloads or work positions.
- The oldest age groups reported in the study had to include people over the age of 50.
- The oldest age groups included at least 20 study subjects.
- Estimates were reported in relation to age group.
- Studies included had to be retrospective, cross-sectional, prospective, or longitudinal.
- Articles published in English, French or Scandinavian languages were considered, with no limitation for the year of publication.

#### Exclusion criteria

- Case studies, case series, case-control studies.
- Studies that did not clearly distinguish between neck- and shoulder pain.
- Studies that did not clearly distinguish between upper limb and shoulder pain.
- Studies that concerned specific clinical populations or specific causes of pain/injuries, including frozen shoulder and osteoarthritis.

### General criteria

The following general criteria were tabulated for each study: first author, year of publication, country of study, mode of data collection (questionnaire/interview/clinical data), type of occupation, final sample size, broad classification of shoulder pain definition, recall periods for pain (point prevalence, 1-year prevalence, lifetime prevalence), number of participants by age category, response rate, and percentages (with *p*-values for trend) or odds ratios with increasing age including confidence intervals.

### Methodological quality

Two reviewers (CH, CLY) independently performed a critical analysis of the included prevalence studies to determine their validity and risk of bias. This was performed using modified criteria (Table 1) based on those from previous prevalence systematic reviews [10–12]. We removed some items of previous quality assessment tools as they were considered not to add value to the assessment of these studies. The items removed were: ‘were data collected directly from the subjects?’, and ‘was the same mode of data collection used for all subjects?’. The checklist uses three methodologic domains with 9 individual criteria for prevalence studies. Overall, these examine representativeness, data quality, and definition of shoulder pain. Any discrepancies in the assessment were reviewed and resolved by consensus. A third author was available in case of disagreements.

**Table 1:** Methodological assessment criteria

A. Is the final sample representative of the target population? (external validity)	
1. At least one of the following must apply in the study: an entire target population, randomly selected sample, or sample stated to represent the target population. 2. Response rate > 80%? If not: a. At least one of the following: reasons for nonresponse described, non-responders described, comparison of responders and non-responders, or comparison of sample and target population.	
B. Quality of the data? (internal validity)	
3. Was the primary objective of the study the collection of data on musculoskeletal pain (including the shoulder) or was it taken from a survey not specifically designed for that purpose? 4. At least one of the following in the case of a questionnaire: a validated questionnaire or at least tested for reproducibility. 5. At least one of the following in the case of an interview: Interview validated, tested for reproducibility, or adequately described and standardized. 6. At least one of the following in the case of an examination: Examination validated, tested for reproducibility, or adequately described and standardized.	
C. Definition of shoulder pain (SP) (internal validity)	
7. Was there a precise anatomic delineation of the shoulder area or reference to an easily obtainable article that contains such specification? 8. Was there further useful specification of the definition of SP, or question(s) put to study subjects quoted such as the frequency, duration or intensity, and character of the pain. Or was there reference to an easily obtainable article that contains such specification? 9. Were recall periods clearly stated: e.g., 1 week, 1 month, or lifetime?	
E. Summary	
10. Item on overall quality and risk of study bias.	

### Data analysis

All prevalence estimates stratified by age were extracted into tables and compared between reviewers for consistency. For two studies, the prevalence estimates had to be measured from charts, as the studies did not provide the exact estimates [13, 14]. This was performed by two blinded authors (CH, CLY).

Similar methodologic quality scores, age categories, definitions of shoulder pain, and type of occupation were considered minimum criteria for pooling of data. However, preliminary analyses showed that the results of the studies could not be statistically pooled as they were not homogeneous for these items. Due to the lack of homogeneity across the included studies and because only 16 of the included studies provided *p*-values for trend or confidence intervals, two of the authors performed case-by-case analysis of the prevalence estimates and odds ratios. The studies were assigned a direction of the estimates for shoulder pain as either ‘increasing’, ‘remaining stable’ or ‘decreasing’ past the age of 50.

Within the table, the included studies were separated into physically active occupations and sedentary occupations. Physically active workers were defined as those occupations that were classified as either ‘labourers’, ‘machinery operators and drivers’, ‘community and personal service workers’, or ‘technicians and trades workers’ according to the Australian and New Zealand Standard Classification of Occupations (ANZSCO) version 1.2. Nurses, nurses’ aides, physical therapists and surgeons were also considered to have physically demanding jobs, despite being officially categorised as ‘professionals’ in the ANZSCO.

Sedentary workers were defined as those occupations that were classified as ‘managers’, ‘professionals’, ‘clerical or administrative workers’, or ‘sales workers’. Although this group of occupations was considered sedentary, most of these workers were considered to likely be exposed at least to some repetitive use of their upper limbs through computer work.

The studies were ordered from high to low based on their methodological quality scores. We had greater confidence in the results of the higher-quality studies and less in those with lower scores, particularly if the poorer studies had conflicting results from the other studies.

## Results

### Study selection

**Fig. 1:**
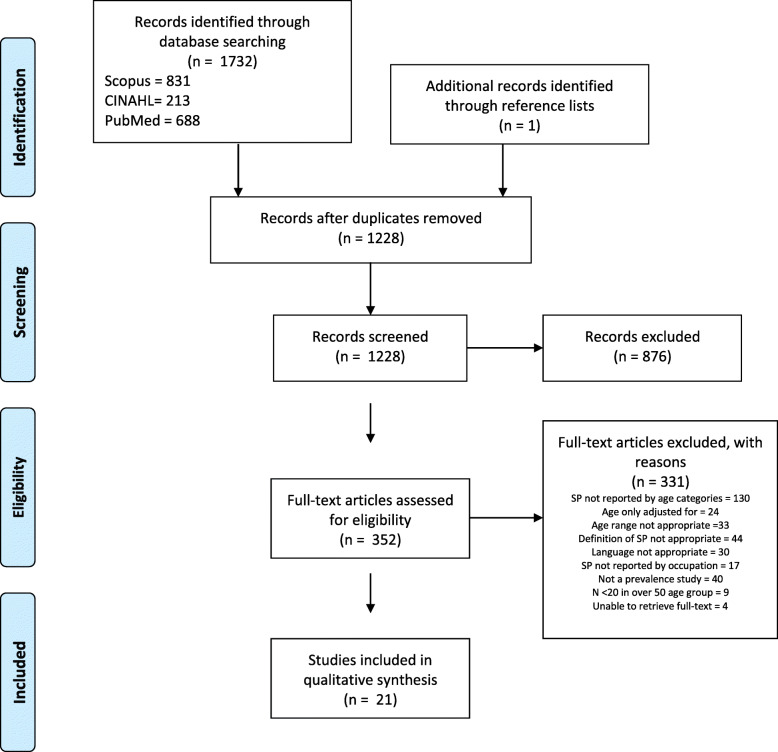
PRISMA flowchart showing the selection process. SP: shoulder pain

By January 2020, the electronic database search had yielded 1228 unique studies (Fig. 1). After abstract and title screening, 352 relevant studies were identified and assessed as full-text articles. One additional potentially eligible study was identified through inspection of reference lists in full-text articles. Initially, 27 studies were considered eligible for inclusion; however, six of these were excluded due to having < 20 participants in the > 50 age category [15–20]. At the end of the screening process, 21 studies were considered eligible for inclusion [13, 14, 21–39]. Three studies did not report the number of participants by age category, but were still included, as the number of the oldest participants were at least 20 based on prevalence estimates [13, 14, 30].

### General study characteristics

In all, 20 of the included studies used either cross-sectional study designs or reported baseline results of longitudinal studies with a total of 40,487 participants [13, 14, 21–28, 30–39]. The final study analysed a clinical database that included cases of acute arm and shoulder conditions [29]. None of the included studies investigated a general population stratified by both age and occupation; instead, the studies investigated one or two occupations. There were 15 studies on non-sedentary occupations, three studies on sedentary occupations, and one study investigated one of each. The general characteristics of each study are identified in Table 2 and are briefly explained below.

**Table 2:** General description and shoulder pain estimates stratified by age of the 21 included studies, grouped by occupation type and ranked by methodological quality

1st Author Year [Reference]	Quality score (/7)	Country of study	Type of occupation (n)	Collection Mode	Definition of shoulder pain	Duration of recall	Age groups (n)	Prevalence (%), Incidence or OR		Direction of estimates^b^
**Physically active occupations**										
Kim 2015 [31]	7	South Korea	Cameramen (166)	Nordic styled questionnaire	Pain for at least one day a month, or for at least seven consecutive days in the past year.	12 months	30–39 (35) 40–49 (66) ≥50 (65)	8.6 13.6 18.5 *p > 0.05		gradient
Freimann 2013 [30]	7	Estonia	Nurses (221)	Self-administered CUPID questionnaire	Shoulder pain (body diagram) lasting longer than a day a during the past year and past month.	12 months	23–29 30–39 40–49 50–59 §	OR: 1 3.2 (0.9–11.9) 5.1 (1.3–19.6) 6.2 (1.4–26.9)		
Armed Forces 2013 [29]	7	U.S.A	Military (NR)	Hospital and outpatient medical records	Several ICD-9 codes for acute and chronic shoulder conditions.	10 year Incidence^*a*^	< 20 20–24 25–29 30–34 35–39 40–44 45–49 ≥50 §	Acute: 13.5 13.7 13.8 12.9 13.6 14.7 15.9 15.8	Chronic: 15.8 19.2 25.2 29.5 41.6 54.7 65.3 71.0	
Moon 2019 [38]	6	South Korea	Food service workers (1581)	KOSHA self-administered questionnaire, history taking by occupational physicians, and physical	Rotator-Cuff Syndrome	12 months	< 50 (591) ≥50 (990) ¥	6.6 10.3 *p > 0.05		
Sombatsawat 2019 [39]	5	Thailand	Farm workers (156)	Clinician administered Nordic Questionnaire	Trouble^ in region of body diagram.	6 months	18–50 (93) 51–65 (63)	OR 1 1.49 (0.61–3.67) *p* = 0.38		
Sekkay 2018 [37]	6	Canada	Truck drivers (123)	ESQ98 (Modified Nordic)	Trouble^ in shoulder region of body diagram.	12 months	< 50 (54) ≥50 (66)	13 24.2 *p > 0.05		
Dianat 2017 [36]	6	Iran	Surgeons (312)	Nordic Questionnaire	Trouble^ in shoulder region of body diagram.	12 months	< 40 (102) 40–50 (106) > 50 (104)	34.3 39.6 46.2 *p > 0.05		
Alrowayeh 2010 [28]	6	State of Kuwait	Physical therapists (222)	Self-administered Nordic Questionnaire	Trouble^ in shoulder region of body diagram.	12 months	20–30 (45) 31–40 (112) 41–50 (27) ≥50 (28)	4.2 7.0 0.9 0.9 *p* = 0.85		=
Guo 2004 [14]	6	Taiwan	Retail industry (18,942)	Questionnaire	Body diagram	12 months	< 18 18–24 25–34 35–44 45–54 55–64 > 64 §	♂ 9.5♀ 12.5 ♂ 10.5♀ 11.0 ♂ 12.5♀ 15.0 ♂ 15.0♀ 16.0 ♂ 18.0♀ 17.5 ♂ 22.0♀ 19.0 ♂ 17.0♀ 17.5 †		
Eriksen 2003 [22]	6	Norway	Nurse’s aides (6485)	Modified Nordic Questionnaire	Trouble^ in shoulder region of body diagram.	2 weeks	< 30 (504) 30–39 (1318) 40–49 (2612) 50–59 (1763) > 59 (283)	39.7 45.1 47.7 49.6 47.0 *p < 0.05		=
D’Agostin 2017 [35]	5	Italy	Nurses (177)	Modified Nordic Questionnaire	Trouble^ in shoulder region of body diagram.	12 months	> 34 (45) 35–54 (110) > 55 (22)	31.1 37.1 45.5 *p > 0.05		
Luime 2004 [24]	5	The Netherlands	Nursing home/ elderly care workers (556)	Nordic Questionnaire	Trouble^ in shoulder region of body diagram.	12 months	< 30 (94) 30–39 (154) 40–49 (192) 50–65 (116)	OR: 1 0.95 (0.47–1.93) 1.72 (0.89–3.29) 1.14 (0.51–2.52)		
Leclerc 2004 [23]	5	France	Assembly line, clothing, food industry (598)	Questionnaire	Body diagram	6 months	≤29 (117) 30–39 (225) 40–49 (203) ≥50 (53)	♂ 34♀ 31 ♂ 32 ♀ 49 ♂ 46 ♀ 51 ♂ 60♀ 65 †		
Backman 1983 [13]	4	Finland	Professional Drivers (1156)	Questionnaire	Self-reported	1 month	30–34 35–39 40–44 45–49 50–54 §	65.0 63.0 68.0 72.0 78.0		
Min 2016 [33]	4	South Korea	Farmers (1013)	KOSHA self-administered questionnaire	Pain (body diagram) for at least one day a month, or for at least seven consecutive days in the past year.	12 months	< 65 (798) ≥65 (215)	43.6 40.5 *p* = 0.41		
Haukka et al. 2006 [25]	4	Finland	Kitchen workers (495)	Self-administered questionnaire	Body diagram	3 months	≤40 (148) 41–50 (185) ≥50 (162)	22 (15.3–28.7) 34 (27.2–40.8) 44 (36.3–51.6) *p* < 0.005		
Ueno 1999 [21]	4	Japan	Construction workers (2289)	Questionnaire	Self-reported	Point	20–24 (107) 25–29 (197) 30–34 (207) 35–39 (236) 40–44 (337) 45–49 (372) 50–54 (295) 55–59 (247) 60–64 (215) 65–69 (76)	11.0 18.0 22.0 24.0 27.0 37.0 40.0 33.0 32.0 29.0 †		
Stucchi 2016 [34]	3	Italy	Retail sector (3359)	Interview	Shoulder region diagnoses (rotator cuff/bicep tendinopathy and subacromial bursitis).	12 months	15–24 (89) 25–34 (623) 35–44 (1372) 45–54 (1061) 55–64 (214)	OR: 0 cases 1 1.85 (1.19–2.86) 3.13 (2.04–4.82) 6.35 (3.87–10.42) *p* < 0.01		
**Sedentary occupations**										
Kalinene 2016 [32]	7	Lithuania	Computer workers (513)	Nordic Questionnaire	Trouble^ in shoulder region of body diagram.	12 months	23–29 (55) 30–39 (91) 40–49 (149) 50–70 (218)	34.5 46.2 50.3 56.4 *p < 0.05		
Janwantanakul 2008 [27]	6	Thailand	Office workers (1185)	Self-administered Modified Nordic Questionnaire	Trouble^ in shoulder region of body diagram.	12 months	> 30 (346) 30–39 (494) 40–49 (262) > 49 (83)	16.0 14.0 19.0 14.0 *p > 0.05		
D’Agostin 2017 [35]	5	Italy	University staff -computer use (185)	Modified Nordic Questionnaire	Trouble^ in shoulder region of body diagram.	12 months	> 34 (63) 35–54 (96) > 55 (26)	22.2 30.2 19.2 *p > 0.05		
Leroyer 2006 [26]	2	France	Administration (759)	Clinician-administered questionnaire	Self-reported	1 week	≤30 (146) 31–40 (249) 41–50 (271) ≥51 (93)	13.7 15.7 25.8 26.9 *p* < 0.01		

*OR* odds ratio, *NR* not reported

^Trouble includes ache, pain, discomfort, or numbness

^a^Incidence rates per 1000 person-years

^b^Direction estimates are comparing those < 50 and those ≥50

§Study does not provide (n) per group

¥Study only included female workers

*p-value only reported > or < 0.05

^†^*p*-value not reported

Studies were published between 1983 and 2019 on populations living in Europe (*N* = 10), Asia (*N* = 7), the Middle East (*N* = 2), and North America (*N =* 2). Of the 21 studies, 19 (90%) were published after 2000. In two studies, all the participants included in the analysis were female. The largest number of age-groups was ten [21], and the smallest was two [33, 37]. One study accessed a clinical database that included almost 200,000 incident cases of acute arm and shoulder conditions [29]. The largest number in the oldest age category was 990 (≥50) [38], and the smallest was 22 in the category (> 55) [35]. No studies were excluded based on methodological quality.

### Methodological quality

The methodological quality of the included studies is shown in Table 3. The quality scores ranged from 2 to 7 out of 7, with four studies achieving a full score [29–32]. The most common deficiencies within the studies were:

- Eleven did not achieve (or report) a representative sample.
- Eight did not use a validated questionnaire, interview, or examination.
- Eight did not achieve > 80% response rate or provide appropriate information regarding non-responders.
- Five did not provide precise anatomical delineation or body diagram and another four did not further specify the definition of shoulder pain.

**Table 3:**
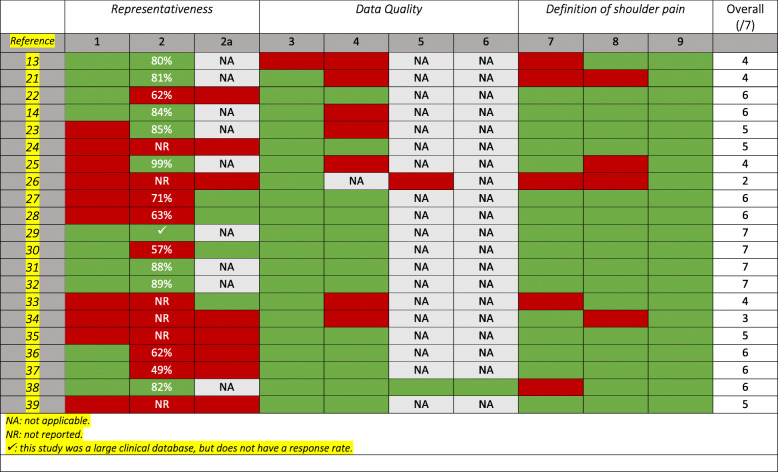
Methodological quality assessment of 21 shoulder pain prevalence studies

### Definitions of shoulder pain

As can be seen from Tables 2, 11 of the studies used the Nordic Musculoskeletal Pain Questionnaire, either in its original form or in a modified version. A further seven studies used either shoulder diagnoses, body diagrams, or provided additional information on the pain, and all reported the recall period(s).

### The direction of estimates by type of occupation

The prevalence of shoulder pain generally increased past the age of 50, with 16 of the 21 included studies reporting higher estimates in older participants. As shown below, the majority of the studies investigating physically active occupations reported shoulder pain prevalence to be higher over the age of 50. Whereas this was less common in those reporting on sedentary occupations.

There was a total of 37,845 participants in 18 studies reporting on physically active workers. The prevalence estimates or odds ratios in those over 50 increased in 14 of these 18 studies [13, 14, 21, 23, 25, 29–31, 34–39], remained stable in two [22, 28] and decreased in one [24]. One study of farmers reported data only for two age groups, using 65 as a cut point and found that shoulder pain reporting decreased in the older group [33]. The prevalence estimates for all studies stratified by age are shown in Table 2.

There was a total of 2642 participants in the four studies reporting on sedentary occupations. Two of these studies had prevalence estimates that continued to increase in people over the age of 50 [26, 32] whereas they decreased in two [27, 35].

## Discussion

### Summary of findings

To our knowledge, this is the first systematic review to assess the prevalence of shoulder pain in occupational groups, stratified by age. The 21 included studies had varying degrees of methodological approaches and quality, for which reasons their data were not suitable to be pooled. Sixteen of the included studies provided *p*-values for age trend, but many did not. Therefore, we performed a visual analysis based on the direction of estimates in age groups. The findings consistently showed that shoulder pain becomes increasingly common over the age of 50 for people who are still in the workforce. This was particularly apparent in studies including occupations defined as physically demanding, but results were conflicting in those studies including sedentary jobs.

However, all of the sedentary occupations included computer work, which requires prolonged use of arms and shoulders with arms stretched out away from the trunk [40]. This may explain why two out of the ‘sedentary’ studies had similar findings to the ‘physically demanding’ studies. Also, one of these two studies rated very poorly on the quality assessment tool, only achieving a total score of one, making the results uncertain.

One study investigating farmers found that shoulder pain did not increase past the age of 65 [33]. This is obviously not consistent with other studies on physically demanding occupations. However, there was no detailed information on work activities past the age of 50, so we could not determine if farmers continue to work into old age or if they stop or slow down soon after 50.

We found decreasing estimates in only one study on physically demanding jobs; aged care workers, which also suggested a decrease in incidence estimates past the age of 50 [24]. However, we have limited confidence in this result as the study did not provide information regarding the representativeness of the study sample. The study also failed to report a response rate and neglected to describe non-responders.

It could be argued that across occupations, and particularly those that are physically demanding, the ‘healthy worker effect’ is likely to be at play. This leaves a ‘healthy worker’ population within that occupation, thus decreasing the prevalence estimates of shoulder pain [41, 42]. It is possible that this ‘effect’ is more marked in some particularly physically demanding jobs, such as farming and some types of nursing. If this phenomenon is at play, these estimates would have underestimated the real impact that physically demanding jobs have on shoulder problems in the elderly [41].

### Comparison to a previous systematic review

The authors of a previous systematic review by Vincent et al. [7] noted that shoulder pain prevalence estimates decrease in the general population over the age of 60–65, despite a continued increase in the presence of rotator cuff pathology. They theorised that one reason for this could be that people in this age bracket with shoulder pathology have either retired, changed occupations or simply are not exposing themselves to as much shoulder demanding activities as in younger age. Our results provide support for this theory, in that those individuals still employed past the age of 50, especially those in physically demanding positions, are more likely to report shoulder pain than those who are younger. However, because our review did not investigate retirees, we are not able to definitively say if retirement would be responsible for a decrease in shoulder pain reporting.

### Methodological considerations of this review

First, as in all systematic reviews, it is not guaranteed that we found all the studies in this area. Nevertheless, we did search PubMed, CINAHL and Scopus using a Medical Subject Heading terms in a search strategy developed with the assistance of a subject librarian. Additional searches via reference lists yielded only one additional study, enhancing our confidence in the completeness of the systematic search strategy.

Secondly, we were unable to identify any extensive population studies that produced prevalence estimates by age and occupation. Such studies could have provided results that may have improved confidence in the outcome, as they would not be so contingent on the healthy worker effect [41]. Furthermore, it is important to acknowledge that the occupation classifications do not take into account potentially relevant factors such as load, repetitive movements, and shoulder elevation. It may also have been worthwhile considering three categories of workers: heavy manual work, physically active non-heavy work, and sedentary work. However, this was not possible due to the number and types of studies included.

The third point relates to our assessment of quality and risk of bias. There are several methods to deal with quality and risk of bias in systematic reviews. Unweighted or weighted marks can be given and added up to a final score using cut-points to identify ‘acceptable’ and ‘unacceptable’ studies. It is also possible to concentrate merely on items that indicate a risk of bias and to estimate the credibility of study results based on these. We opted for a score and simply ordered the studies by this score, from high to low, acknowledging that there is probably no definite cut-off point. Instead we had more trust in those studies with better methodology scores, particularly when results went in the same direction.

Fourthly, due to the statistical and methodological heterogeneity of the included studies, it was not possible to perform a meta-analysis, which could have provided us with actual estimates. Instead, we reported our findings as either ‘increasing’, ‘remaining stable’ or ‘decreasing’. In our analyses, we did not test for statistically significant changes between age groups, because these groups were often very small and small group sizes may lead to a type two statistical error. It is important to note that some of the observed differences across age groups were statistically significant and others were observed tendencies which were subjective in nature. The decisions for the direction of estimates were made independently by two review authors, however due to the nature of the visual analysis may contain biased interpretations of the results. Some directions of estimates were not based on statistically significant results. Nevertheless, the fact that most estimates went in the same direction strengthened our interpretation.

### Methodological considerations of the included studies

Several studies ultimately had to be excluded during the screening process because they did not describe age demographics by category. This not only made it impossible to establish the direction of prevalence estimates with age but also meant that even in those studies that had reported prevalence by age, it was not possible to know how many were actually in the over 50 brackets. Descriptive statistics of population samples should provide the number of participants in age categories so that it is possible to visualise the spread of the sample. Furthermore, we could not include the age range or maximum age for those studies that were included, because it was rarely provided.

Our review identified several methodological flaws in the included studies. Just over half of the studies failed to achieve or report a representative study sample, with a variety of sample sizes. Six studies did not achieve a response rate of at least 80% and a further six did not even report the response rate. The low response rate, coupled with a lack of description of non-responders, left these studies at risk of selection bias and ultimately impacted our confidence in the representativeness of the sample. Other authors have astutely used a confidence interval approach or wave analysis to determine if those who responded quickly to a survey in the first “wave” were different in their pain reporting to those who responded at a later time (second or third waves). If they do, it suggests that non-responders are also likely to be different in some way [43].

The majority of included studies used a variation of the Nordic Musculoskeletal Pain Questionnaire, but in those that did not, there were unclear definitions of shoulder pain [44]. Some failed to provide an anatomical delineation of shoulder pain either via description or a body diagram. Others failed to provide further information regarding what constitutes a shoulder pain episode, such as severity, duration, and character. This could impact on the accuracy of the pain reporting, as study subjects would have to know what was meant by ‘shoulder’ and by ‘pain’.

Finally, the included studies did not consider the role of other activities from daily life, leisure, or sport. While it was not our aim to investigate these other activities, they could be important modifying factors in the onset and development of shoulder pain. This could be a factor in helping to explain the conflicting results, particularly from the sedentary groups.

## Conclusion

The results of this systematic review indicate that shoulder pain continues to be prevalent in older populations that are still working, and particularly if their work is physically active or, at least, involves the use of their upper limbs.

Future research of this type should include a clear description of the anatomical delineation of the shoulder and should report pain patterns and severity by age and occupation. In particular, it is essential to separate transient minor episodes of pain from chronic disabling pain that may require management by a health professional. In survey studies with a low response rate, authors should consider performing a wave analysis to examine potential non-response bias.

### Implications for physiotherapy practice

Shoulder pain appears still to be prevalent in older populations that are still working, and particularly if their work is physically active or, at least, involves use of their upper limbs. This may aid clinicians in making a prognosis on the evolution of shoulder pain in active and sedentary works and ultimately impact clinical decision making.

## Supplementary Information

**Additional file 1.** PRISMA Checklist. The completed PRISMA checklist.

**Additional file 2.** Search Strategy. The complete search strategy for the systematic review.

## Data Availability

Data sharing is not applicable to this article as no datasets were generated or analysed during the current study.
