# Correction to: Shoulder pain prevalence by age and within occupational groups: a systematic review

**DOI:** 10.1186/s40945-021-00127-w

**Published:** 2022-01-10

**Authors:** Christopher J. Hodgetts, Charlotte Leboeuf-Yde, Amber Beynon, Bruce F. Walker

**Affiliations:** 1 Discipline of Chiropractic, College of Science, Health, Engineering and Education, Murdoch University, Perth, Western Australia Australia; 2 Centre for Molecular Medicine and Innovative Therapeutics, Health Futures Institute, Murdoch University, Perth, Western Australia Australia; 3 Institute of Regional Health Research, University of Southern Denmark, Odense C, Denmark

**Correction to: Archives of Physiotherapy 11, 24 (2021)**

**https://doi.org/10.1186/s40945-021-00119-w**

Table 3 of the original article [1] included coloured cells that were incorrectly removed during typesetting.

The correct table is shown here below and has already been included in the original article.

**Table 3:**
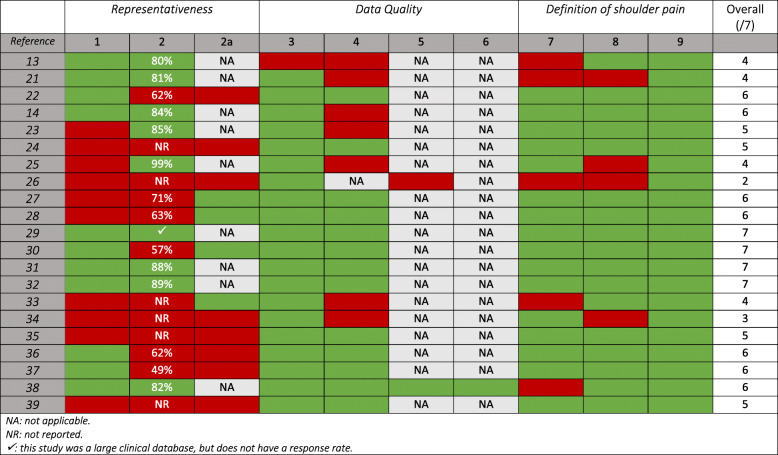
Methodological quality assessment of 21 shoulder pain prevalence studies

Na: not applicable

NR: not reported

✓: this study was a large clinical database, but does not have a response rate
