# Supplementary material for: Shoulder pain prevalence by age and within occupational groups: a systematic review
Source: Arch Physiother. 2021 Nov 4;11:24. doi: 10.1186/s40945-021-00119-w (PMC8567712; doi:10.1186/s40945-021-00119-w)
Supplement: Supplementary file 2 — Additional file 2. Search Strategy. The complete search strategy for the systematic review. [file 40945_2021_119_MOESM2_ESM.docx]

**Appendix 1:**

P: Global Adult population (particularly over 50 years)

I: Occupational factors

O: Prevalence/incidence of shoulder pain

**Key Words:**

| “Shoulder Pain” | Prevalence | Age | Occupation |
| --- | --- | --- | --- |
|  | Epidemiology  Incidence  Rate  Occurrence |  | Work  Sport  Workload  Ergonomics  Position |

**Databases:**

CINAHL

PubMed

Scopus

**Search Strategy**

**CINAHL Search Strategy**

shoulder N3 Pain AND ( epidemiology OR incidence OR prevalence OR rate OR occurrence ) AND age AND ( occupation OR work OR sport OR workload OR position OR ergonomics )

Results: **213 (06/02/2020)**

***NB: “****Shoulder N3 Pain” retrieves records that contain phrases with up to three words between shoulder and pain, in either direction. E.g. “shoulder pain” or “pain in the shoulder”.*

**PUBMED Search Strategy**

((("shoulder pain"[MeSH Terms] OR ("shoulder"[All Fields] AND "pain"[All Fields]) OR "shoulder pain"[All Fields]) AND (("work"[MeSH Terms] OR "work"[All Fields]) OR ("workload"[MeSH Terms] OR "workload"[All Fields]) OR ("occupations"[MeSH Terms] OR "occupations"[All Fields] OR "occupation"[All Fields]) OR ("sports"[MeSH Terms] OR "sports"[All Fields] OR "sport"[All Fields]) OR ("ergonomics"[MeSH Terms] OR "ergonomics"[All Fields]) OR position[All Fields])) AND (("epidemiology"[Subheading] OR "epidemiology"[All Fields] OR "epidemiology"[MeSH Terms]) OR ("epidemiology"[Subheading] OR "epidemiology"[All Fields] OR "prevalence"[All Fields] OR "prevalence"[MeSH Terms]) OR ("epidemiology"[Subheading] OR "epidemiology"[All Fields] OR "incidence"[All Fields] OR "incidence"[MeSH Terms]) OR ("epidemiology"[Subheading] OR "epidemiology"[All Fields] OR "occurrence"[All Fields] OR "epidemiology"[MeSH Terms] OR "occurrence"[All Fields]) OR ("J Rehabil Assist Technol Eng"[Journal] OR "rate"[All Fields]))) AND ("Age"[Journal] OR "Age (Omaha)"[Journal] OR "Age (Dordr)"[Journal] OR "Adv Genet Eng"[Journal] OR "age"[All Fields])Results: **688 (06/02/2020)**

**Scopus Search Strategy**

( TITLE-ABS-KEY ( shoulder  AND pain )  AND  TITLE-ABS-KEY ( epidemiology  OR  incidence  OR  prevalence  OR  rate  OR  occurrence )  AND  TITLE-ABS-KEY ( age )  AND  TITLE-ABS -KEY ( occupation  OR  work  OR  sport  OR  workload  OR  position  OR  ergonomics ) )

Results: **831 (06/02/2020**
